# Drug carriers based on highly protein-resistant materials for prolonged *in vivo* circulation time

**DOI:** 10.1093/rb/rbv003

**Published:** 2015-05-21

**Authors:** Ruiyuan Liu, Yan Li, Zhenzhong Zhang, Xin Zhang

**Affiliations:** ^1^National Key Laboratory of Biochemical Engineering, Institute of Process Engineering, Chinese Academy of Science, Beijing 100190, People’s Republic of China, ^2^School of Pharmaceutical Sciences, Zhengzhou University, 100 Kexue Avenue, Zhengzhou 450001, People’s Republic of China and ^3^University of Chinese Academy of Sciences, Beijing 100049, People’s Republic of China

**Keywords:** long circulation, nonspecific protein adsorption materials, drug carrier, surface modification

## Abstract

Long-circulating drug carriers are highly desirable in drug delivery system. However, nonspecific protein adsorption leaves a great challenge in drug delivery of intravenous administration and significantly affects both the pharmacokinetic profiles of the carrier and drugs, resulting in negatively affect of therapeutic efficiency. Therefore, it is important to make surface modification of drug carriers by protein-resistant materials to prolong the blood circulation time and increase the targeted accumulation of therapeutic agents. In this review, we highlight the possible mechanism of protein resistance and recent progress of the alternative protein-resistant materials and their drug carriers, such as poly(ethylene glycol), oligo(ethylene glycol), zwitterionic materials, and red blood cells adhesion.

## Introduction

When it comes to the *in vivo* application of drug carriers, the long-circulating ability is the most important property which could enhance the accumulation of drug in the target site. However, nonspecific protein adsorption is the first step and the proteins in the blood are intended to interact with drug carriers especially the cationic drug carriers following intravenous injection [1]. Nonspecific protein adsorption is essentially a complicated process that is controlled by both kinetics and thermodynamics. The adsorption process tends to be irreversible due to the denaturation of the protein [2, 3]. The presence of nonspecific adsorption accelerates the blood clearance of drug carriers due to the recognition by the reticuloendothelial system (RES), particularly the hepatic Kupffer cells [4].

The nonspecific protein adsorption in blood such as fibrinogen and clotting enzyme results in the aggregation of drug carriers, which accelerates the clearance of drug carriers and reduces the plasma clearance half-time of drugs. Additionally, the recognition of aggregative drug carriers by RES changes the tissue distribution of the drug carriers and the accumulation of drug carriers in hepatic Kupffer cells is enhanced, which reduces the drug accumulation in the target site [5, 6]. Therefore, it is an important requirement of the drug carriers to resist the nonspecific protein adsorption to extend the circulation time for systemic intravenous and leave enough time for the carriers to interact with the target tissue and cells.

Accordingly, an important challenge to biomaterials is the prevention of nonspecific protein adsorption on surfaces. To achieve this, the drug carriers need to be coated by highly protein-resistant materials to prolong the circulation time and facilitate the targeted accumulation of drug carriers. Generally, it is believed that protein-resistant materials could prevent nonspecific protein adsorption due to their hydrophilic interaction or static electric field [7]. Therefore, the capability to resist nonspecific adsorption is an essential prerequisite and the material should share the following characteristics such as hydrophilic and net charge neutral. Moreover, it is worth mentioning that the polymer coating may hinder the drug release and the cellular uptake behavior of the drug-loaded carriers, which lead to a challenge to the therapeutic efficiency of the drug molecules [8, 9]. Therefore, we must take this factor into consideration when the highly protein-resistant materials were selected. Recently, many attempts have been made to explore various types of functional materials, which have been shown to be highly resistant to nonspecific protein adsorption.

## Synthetic Materials

### Poly(ethylene glycol)

**Figure 1:**
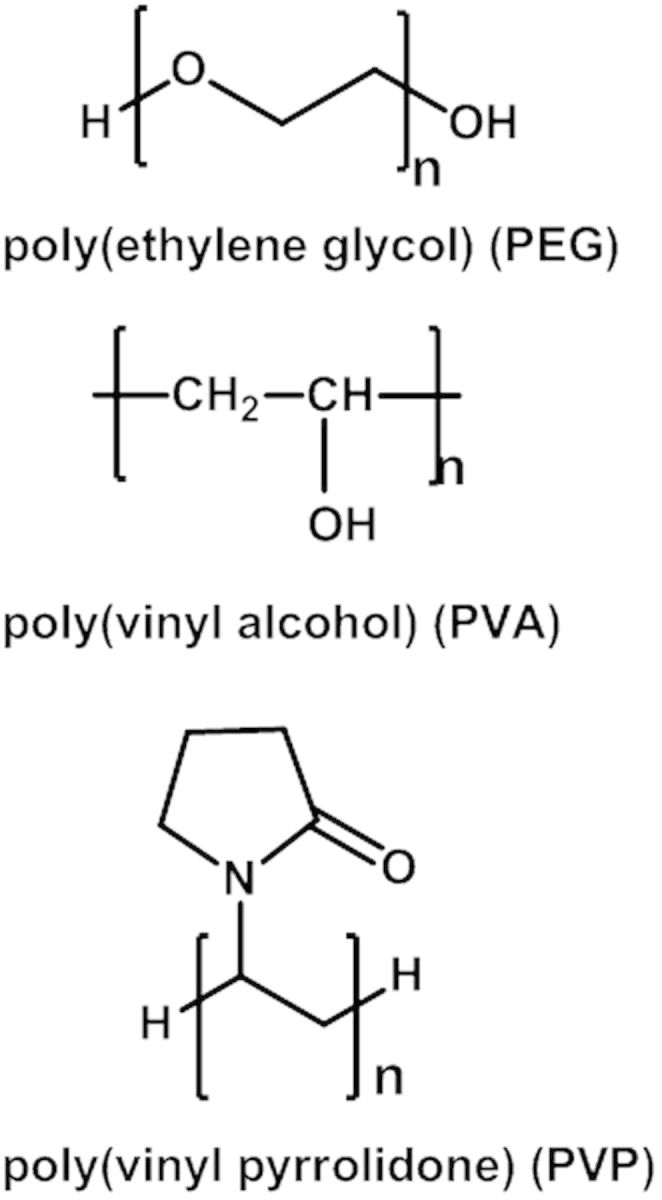
Chemical structure of nonionic polymeric compounds.

The most commonly used protein-repelling material is water soluble polymer, such as poly(ethylene glycol) (PEG), poly(vinyl alcohol), polyethyloxazoline, and poly(vinyl pyrrolidone) or their derivatives (Fig. 1) [10, 11]. Once these polymers are attached onto drug carriers, they all could reduce the amount of protein adsorption. Among these hydrophilic polymers, the performance of PEG is the best [12]. Therefore, PEG is the most popular strategy and it has been extensively studied to modify the surface of drug carriers. PEG is a water soluble and electrically neutral polyether which has been widely used as a coating for drug carriers and the distinctive properties of PEG have attracted the research interests of the chemists. Much effort has been made to theoretically elucidate the intrinsic relationship between the structure and special properties of PEG [13, 14].

Studies during the past two decades have confirmed that the modification of drug carriers with long-chain PEGs (the molecular weight of PEG ≥2000 Da) could significantly reduce the nonspecific adsorption with the protein in blood [15, 16]. Although the kinetic and thermodynamic origin of the protein resistance of PEG is still debated, it is often explained by a ‘steric’ repulsion model. In this model, a steric exclusion effect is considered as one of the reasons for long-chain PEG polymers to resist nonspecific protein adsorption. It is believed that PEG chains could bind with water through hydrogen bond, which leads to a barrier around the PEG chains [17, 18]. Besides, PEG is also a substance with little toxicity which is FDA approved and it has been widely used for modification of protein and peptide drugs for *in vivo* drug delivery. Moreover, PEGylation could be obtained by different methods such as physical adsorption and covalent graft [19].

PEGylated drug carrier delivery systems hold great promising applications for disease therapy and a considerable number of researchers have focused on to the modification of PEG [20, 21]. Perrault *et al.* [22] coated gold nanoparticle cores of five different sizes with PEG of three different molecular weights and tested the circulation time of the PEGylated gold nanoparticles in a mouse model. Dixit *et al*. [23] also utilized protein repellence capability of PEG to stabilize the drug nanoparticles.

Nevertheless, PEG could be decomposed owing to its rapid auto-oxidation in the presence of oxygen and transition metal ions [24]. In addition, the property of protein adsorption resistance was only observed for the room-temperature stable hydrated phase, and not for the high-temperature dehydrated amorphous phase. When the ambient temperature was above 35°C, PEGylation was found to lose the resistance to protein adsorption [25]. Moreover, it has been demonstrated that PEG chains have a large range of motion, and it may leave free spaces between each other, which could also lead to protein adsorption. All these shortcomings accelerate the drug eliminate after long-term exposure to biological fluids, which significantly limit the application of PEG *in vivo*.

Although PEGylation is believed to be beneficial for systemic circulation, it also suppresses the electrostatic cellular interaction and uptake of the drug carriers, thereby dramatically reducing their biological activity [9]. Our group has also proved that PEGylated neutral liposomes have no interaction with the cells by labeling the drug and lipid respectively via real-time tracking. As a result, the ability of drug internalization into cells of the PEGylated carriers will be adversely affected and the long-circulating stealth liposomes typically accumulate predominantly within tumor extracellular space without cell internalization [26]. Similarly, Remaut *et al.* [27] observed that PEG chains caused a dramatic decrease in transfection efficiency by inhibiting the endosomal escape of the cationic liposomes.

**Figure 2:**
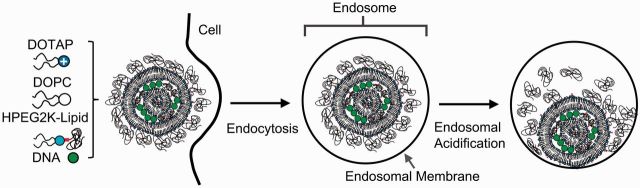
Schematic depiction of the intracellular processing of cationic liposome–DNA complexes stabilized with the low pH-sensitive HPEG2K lipid.

To overcome the barrier of PEGylation, several strategies have been designed to modify the PEG shield. Recently, Chan *et al*. [28] prepared an acylhydrazone-based acid-labile PEG lipid (HPEG2K lipid, PEG MW 2000) whose PEG chain will be cleaved from the tail at lower pH of late endosomes as shown in Fig. 2. Herein, the complexes will be sterically stabilized in the physiological condition and lose their PEG shell in the endosomes. Moreover, Harashima *et al.* [29] have reported a PEG-Peptide-DOPE ternary conjugate which is modified to the drug carrier by a matrix metalloproteinase (MMP)-sensitive group. As the MMP is specifically expressed in tumor extracellular matrix, PEG was removed from the carriers to enhance the endosomal escape in the tumor cells.

Although the outstanding long-circulating property of PEG-containing materials has been recognized for a long time, recent paper reported that repeated injections of the PEGylated liposomes lost their sustained circulation characteristics after a certain time in the same animal [30, 31]. This frustrating effect is known as the ‘accelerated blood clearance (ABC)’ phenomenon, which could compromise the applicability of PEGylated liposomes in many clinical settings. Recent studies have suggested that PEGylated drug-loaded liposomes are intended to stimulate the spleen to produce anti-PEG IgM after the first dose, which selectively recognizes and binds to PEG of the second dose of liposomes and subsequently activates the complement system, causing rapid elimination and enhanced hepatic uptake [32]. Furthermore, it is worth noting that this effect could also be triggered after the administration of PEGylated cationic liposomes [33], polymeric nanoparticles [34], and polymeric micelles containing nucleic acids [35]. The immune response decreases the drug accumulation in the diseased region and presents a tremendous impact on the clinical application of liposomal formulations. In addition, it should be specially mentioned that when the loaded drug is cell cycle nonspecific agents, such as doxorubicin, the ABC phenomenon would be abrogated resulted from the reduced production of anti-PEG IgM by the inhibition of splenic B cells proliferation.

To abrogate the induction of ABC phenomenon, several attempts have been applied by changing the physicochemical properties of PEGylated drug carriers. Xu *et al.* [36] have linked PEG to lipids by ester bonds, which could be cleaved gradually in the blood circulation to avoid or alleviate the ABC phenomenon. Compared with the uncleavable PEG lipids, only a slight or no ABC phenomenon was induced by repeated injection of the cleavable PEG-lipids modified liposomes. However, this approach was accompanied by sacrificing the long-circulation property of PEG at the same time, which would negatively affect the therapeutic efficacy of drugs. Accordingly, it is still of great interest to search for appropriate solution or alternative protein-resistant materials other than PEG.

### Oligo(ethylene glycol)

In the previous studies, it is generally accepted that water at the oligo(ethylene glycol) (OEG)–protein interface plays a very important role in the resistance of OEG to protein adsorption. OEG-terminated alkanethiolate self-assembled monolayers (SAMs) were also found to resist protein adsorption from simple protein solutions. Recent studies have shown that the ability of these ethylene glycol (EG) SAMs to resist the nonspecific protein adsorption depends on their surface packing density which affects the hydration of EG chains [37].

The ‘water barrier’ theory is often used to interpret the protein resistance behavior of OEG-SAM. Surface hydration is generally considered as the key to its resistance to nonspecific protein adsorption for short OEG groups. Proteins could not be adsorbed on the OEG surface because there is a layer of tightly bound water molecules around the OEG chains. Therefore, the protein resistance of OEG-SAMs is mainly due to the difficulty in dehydrating both EG chains/segments and proteins. However, for long-term application, OEG is also proved to be susceptible in the presence of oxygen and transition-metal ions as well as PEG. Moreover, the potential toxicity of the OEG-SAM also limits its application *in vivo*.

## Zwitterionic Materials

**Figure 3:**
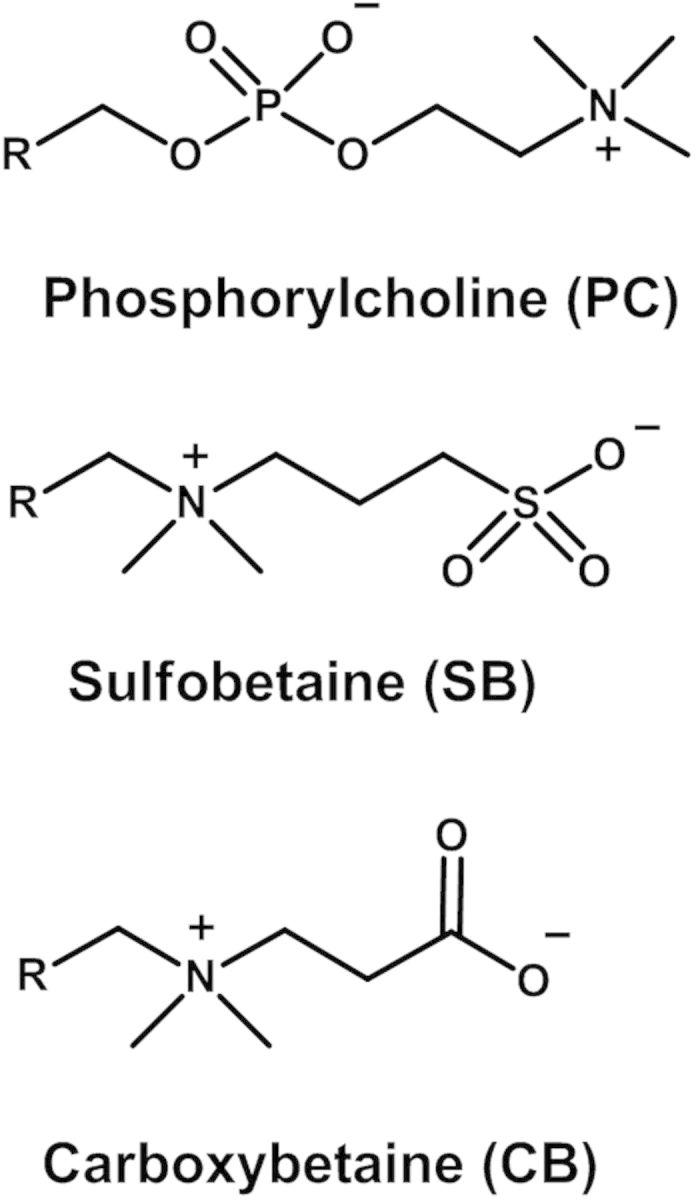
Chemical structures of zwitterionic molecules.

Over the past decade, zwitterionic polymers have attracted huge attention in the field of biomaterials due to their excellent protein adsorption resistance. These zwitterionic materials present outstanding biocompatibilities and have been emerged as a new class of materials for constructing long-circulating nanoparticles [38]. Within the class of zwitterionic materials, phosphorylcholine (PC), carboxybetaine (CB), and sulfobetaine (SB) have been the three most widespread ones (Fig. 3). These materials are generally named after the anionic moiety and contain both anionic and cationic charges in the same unit, which are uniformly mixed with balanced charge. Betaines were used as lubricant oil additives, emulsifying agents, fungicides, and fire-resistant polymers, but we have found that they were also excellent protein resistance polymers applied to long-circulation carrier. In addition, it is believed that the cell uptake of drug carriers with zwitterionic material relatively easily because of adsorptive interactions with the cell membrane. Therefore, polymers based on zwitterionic molecule show promising practical application in drug and gene delivery with excellent transfection efficiency and low cytotoxicity [39].

**Figure 4:**
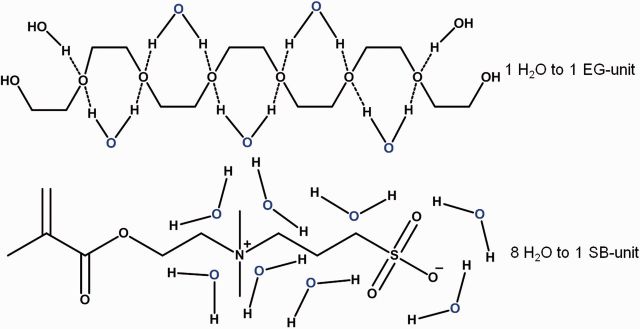
Schematic illustration of the hydration process of both PEG and PSB polymers.

Although hydrophilic and neutral OEG or PEG forms a hydration layer via hydrogen bonds, zwitterionic molecules can achieve stronger hydration by strong ionic salvation. It has been demonstrated that zwitterionic materials lead to a strong repulsive force to protein at specific separation distances because it could form a hydration layer via electrostatic interaction with water molecule [40]. Moreover, Wu and Chen [41] observed that there are ∼8 water molecules bounded on one SB group by differential scanning calorimetry, which is far more than only one bound water molecules on one EG unit, as shown in Fig. 4. Moreover, zwitterionic polymer has been used for the surface modification of nanoparticles like silver [42], silica [43], and gold [44] to enhance the biocompatibility and prolong the blood circulation time of nanoparticles.

### Phosphorylcholine

PC, which is a zwitterionic polar group of phospholipids, has been found to be a promising alternative highly protein-resistant material and presents no negative effect on epithelial cell migration. Biomimetic materials contain PC group, which was demonstrated to be nonthrombogenic and it is a delicate composition and structure existing in the outer membranes of most cells. Similar to PEG, zwitterionic PC has also been shown to have a strong influence on interfacial water molecules [45]. The protein resistance mechanism of PC-based polymers is often explained with a ‘steric repulsion’ model similar to PEG. However, when it comes to drug delivery, the PC group itself has no direct interaction and the function to induce the internalization and endosomal escape, because the PC group is a zwitterionic extremely hydrophilic group with total electrically neutral state [46].

In the previous studies, Ishihara *et al.* [47] first reported that PC modified drug nanocarriers. Over the past two decades, 2-methacryloyloxyethyl phosphorylcholine (MPC), which was designed by a methacrylate monomer with a phospholipid polar group, has been shown to significantly reduce protein adsorption compared with relevant controls. Ishihara *et al.* [48] reported a few MPC-based copolymers which were used to improve the biocompatibility of membranes through the coating or blending methods and they found that the polymers have a stronger ability to hold water molecules than the other groups.

However, Chim *et al.* [49] have reported that the water molecules around hydrated MPC layer promoted the weakening of DNA condensation ability. It was demonstrated that MPC-containing copolymer adversely influenced the transfection efficiency. Moreover, it was confirmed that PC-based materials did not match the excellent protein-resistant property of PEG although they are believed to be more biocompatible. In addition, it was found that PC-based monomers are generally difficult to synthesize and handle, which is not in favor of application [50]. Most importantly, further studies have suggested that PC-based polymers lack long circulation stability, which was attributed to the tendency of hydrolysis of phosphoester groups [51].

### Polysulfobetaine

Because of the disadvantages of PC, as mentioned above, it has been desirable to seek alternative zwitterionic materials which have protein-resistant property except PC-based materials. It has been demonstrated that SB is another important zwitterionic molecule and the polymers of SB have shown effective protein-resistant ability [52]. Polysulfobetaine (PSB) belongs to polybetaines, in which both cationic and anionic groups are on the same monomer residue. Moreover, SB is easier to synthesize and more readily available compared with PC and the structure of SB is similar to that of taurine betaine, which plays an important role in numerous physiological functions. Over the past decade, SB mostly was utilized to improve antifouling properties by grafting to various materials and only a few researches were focused on the drug carriers [53, 54]. In our previous work, it has been demonstrated that SB copolymer could improve cellular uptake and gene transfection efficiency to a greater extent of cationic polymers compared with PC copolymers [55].

However, it has been demonstrated that the solubility of PSB strongly depends on the molecular weight, pH, and ionic strength of the solvent, which is exactly different from the other zwitterionic polymers. As a result, the physical properties should be considered well when PSB was designed to form a drug carrier. Even more frustrating is that, although SB is easy to synthesis, it is short of promising functional group for ligand modification, which negatively affects the further application of the SB-based materials [56]. Moreover, Zhang *et al.* [57] have compared the protein resistance of two copolymers containing PC or SB moieties, respectively. It has been shown that in all cases the polymer coating reduced protein adhesion, but the SB-based copolymer was even not as effective as PC-containing materials at protein adsorption resistance and cell adhesion. Many studies were focused on grafting other functional groups to SB-based materials or changing their surface packing. For example, Chang *et al.* [58] prepared three well-defined diblock copolymers containing PSB with poly(propylene oxide) as a hydrophobic moiety, and the results showed the excellent protein-resistant property of PSB. All these reported weaknesses of PSB inhibit the progress of PSB and it is desirable to develop better zwitterionic materials which are more convenient to the application of drug delivery system.

### Polycarboxybetaine

Compared with other zwitterionic materials, CB might be the most popular zwitterionic molecules recently. CB groups are more attractive because they have many unique properties, such as extraordinary stability, easy preparation, and excellent protein-resistant properties. CB groups not only could highly resist nonspecific protein adsorption, but also have abundant functional groups which are very convenient for the immobilization of biological ligands via EDC/NHS chemistry [59]. Moreover, compared with the other zwitterionic polymers such as PSB, which remains zwitterionic over a wide range of pH, polycarboxybetaine (PCB) zwitterionic monomer can be rendered cationic by lowering the pH of the aqueous medium. And this pH-responsive property can significantly alter rheological properties of PCB polymers. Therefore, PCB-based polymers present the unique ability that they could highly resist nonspecific protein adsorption at the physiological pH of 7.4, and could be protonated at acidic environment which accord with the endosomal pH value. So it was believed that PCB is a class of zwitterionic materials with the characteristic of pH sensitivity [60]. Moreover, the abundant carboxylate anions of PCB make it very convenient for the modification of targeting ligands, therapeutic drugs, and diagnostic labels. All these characteristics make PCB to be a very promising polymer for ‘theranostics’, biomedical, and engineering applications.

It has been confirmed that PCB-coated carriers had long-term stability in human blood serum and very useful for their protein-resistant property [61, 62]. In a previous study, Cao *et al.* [63] reported that nanoparticles formed by PLGA-PCB block copolymers were superior to PEGylated PLGA because of their extraordinary stability, easy processing, and multifunctionality. Wang *et al.* [64] successfully synthesized biomimetic hyperbranched copolymer HBPO-PCB and focused on the targeted delivery to tumor to investigate *in vivo* application. All these results showed that PCB-based materials were a class of candidate carrier for targeted drug delivery.

Recently, our group has modified DSPE lipid with zwitterionic PCB polymer by means of atom transfer radical polymerization and applied DSPE-PCB*_n_* in cationic liposomes modification for siRNA delivery. DSPE-PCB_20_ cationic liposome/siRNA complexes had the excellent serum stability, which was comparable with that of DSPE-PEG 2000. Furthermore, the carboxyl acid groups of PCB could be protonated in endosomes, resulting in accelerated endosomal escape of siRNA and enhanced gene silencing efficiency. The results demonstrated that DSPE-PCB cationic liposomes could be used as an excellent siRNA delivery carrier [60]. Moreover, we conjugated the pH-sensitive PCB with camptothecin (CPT), a traditional chemotherapeutic drug through inducing tumor cells apoptosis, through pH and esterase-sensitive ester bond to form a CPT prodrug. The prodrug-based cationic liposomes were then constructed for drug and siRNA codelivery. With a dual sensitive function of CPT-PCB/siRNA lipoplexes, the system enabled a temporally controlled release of two drugs, which proved to be a promising delivery system. In addition, it was also proved that PCBylation liposomes perform better to stabilize liposomes as a protective layer to extend the blood circulation of the drug carrier [65]. Therefore, it is believed to be a promising alternative long-circulation material to replace PEG.

**Figure 5:**
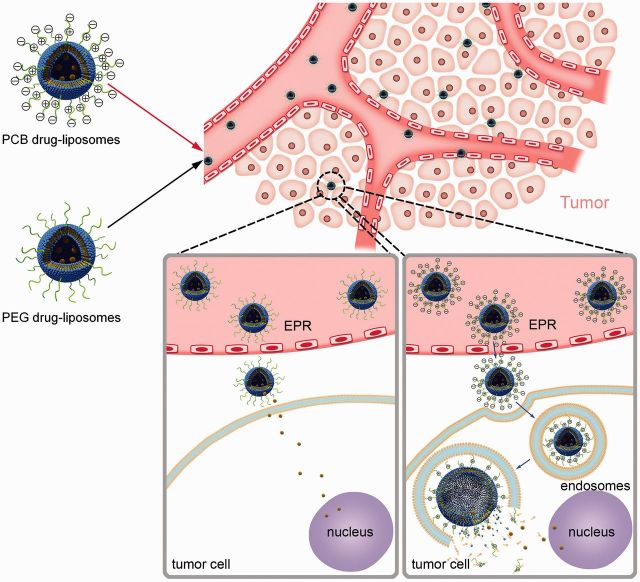
Schematic diagram of the cellular uptake behavior of the DSPE-PCB and DSPE-PEG drug-loaded liposomes.

We have confirmed that PEGylation negatively affect the cellular interaction and uptake of the drug carriers. To investigate the cellular uptake behavior of PCBylated carriers, our group has examined the intracellular trafficking of both PEG and PCB drug-loaded liposomes. The results indicated that PCBylated liposomes could be internalized via endocytosis, while PEGylated liposomes might be internalized via diffusion after extracellular release (Fig. 5). Therefore, it was inferred that the different chemical structure which has positive charge groups is the primary cause [26].

**Figure 6:**
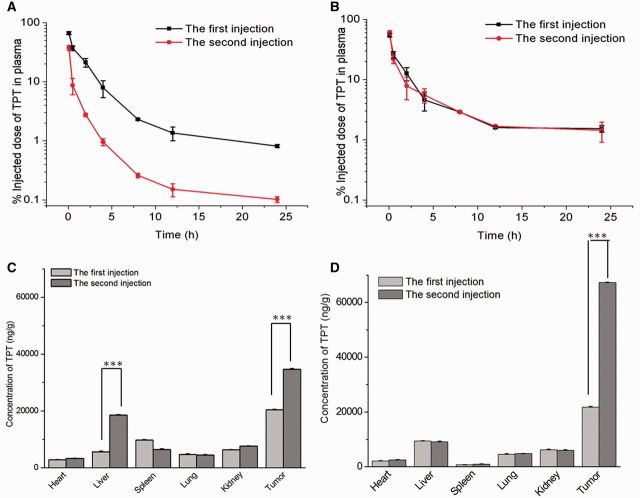
Blood clearance and biodistribution in rats and mice, respectively. (A) and (C) for DSPE-PEG TPT liposomes. (B) and (D) for DSPE-PCB TPT liposomes. The TPT concentration was 7 mg/kg and the time interval was 5 days. *P < 0.05, **P < 0.01, ***P < 0.005 (*n* = 3).

**Figure 7:**
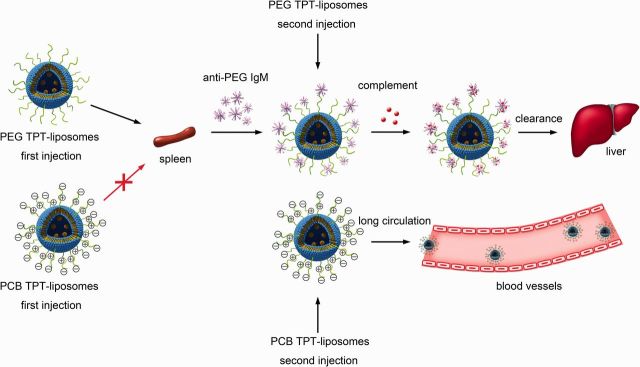
Schematic illustration of the ABC process of the PCBylated and PEGylated drug-loaded liposomes.

We have mentioned that PEGylation liposomes may induce ABC phenomenon at certain time intervals in the same animal, which markedly reduces the bioactivity of the drugs. But the issue whether PCB polymers show this disadvantage has not been investigated. To answer this question, our group developed a DSPE-PCB modified topotecan (TPT) liposomes drug delivery system and explored the pharmacokinetics of the drug carrier with the blood and tissues for two dose (time interval of 5 days). As the blood clearance profile shown in Fig. 6A, DSPE-PEG TPT liposomes triggered a rapid blood clearance of the second dose. But for DSPE-PCB TPT liposomes, the pharmacokinetic profile of the second dose was basically the same as the first dose (Fig. 6B). Moreover, when A549 tumor-bearing nude mice were injected with DSPE-PEG liposomes, the liver uptake of the second dose was three times that of the first injection (Fig. 6C). However, after the second dose, the liver accumulation was unchanged of DSPE-PCB liposomes (Fig. 6D). Thus, it was demonstrated that DSPE-PCB modified liposomes would not only extend the blood retention but also avoid ABC phenomenon which PEGylation drug carriers induced. The schematic illustration of the ABC process of the PCBylated and PEGylated drug-loaded liposomes was shown as Fig. 7. In addition, the modification of DSPE-PCB could also avoid the production of anti-PCB IgM [26]. Recently, our group has demonstrated that DSPE-PCB modified siRNA liposomes could avoid the ABC phenomenon as well [66].

## Natural Materials

### Red blood cells

Over the past two decades, a number of biocompatible polymeric materials have been extensively applied in intravascular drug delivery to achieve the ability of long circulation [67]. But the prolonged circulation time of compatible polymers is generally on the order of a few hours. In addition, the method is only effective to relatively small particles about 200 nm. And it has been reported that the protective function of surface modification is reduced with repeated injections [68]. To solve this problem, it is still urgent to find another method to achieve half-time prolonged of larger size drug carriers.

**Figure 8:**
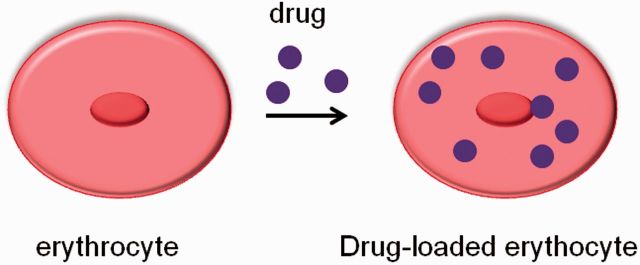
Noncovalent adhesion of drugs with erythrocytes.

Recently, it has been demonstrated that the circulation time of nanoparticles can be prolonged by their noncovalent adhesion on red blood cells (RBCs), as shown in Fig. 8, which are the main carriers of oxygen to cells and tissues of the body and constitute the largest population of blood cell [69]. This strategy was based on mammalian pathogens such as eperythrozoonosis and hemobartonella (0.2–2 μm), and it has been found that the pathogens could bind to the RBC surface and persist in circulation for several weeks. This unique property of RBC offers a promising candidate for systemic delivery of drug carriers in a widely range sizes. Based on this consideration, a biocompatible, nonimmunogenic cellular drug delivery system with a long life-span in circulation was developed and it has been found that the appropriate sizes of particles ranged from 100 nm to 1.1 μm [70]. Erythrocytes are very easy to get and it is realizable to encapsulate large amounts of drugs in a small volume of cells. Samir *et al.* [71] further investigated the strength of binging between the particle and the RBC and it was demonstrated that adhesion strength could be increased via lectins, peptides, antibodies, or covalent attachment.

RBC is believed to be biological origin carriers, and compared with other drug carriers, however, it was indicated that this method may present strong variability and lesser standardization in the preparation [72]. Moreover, it is still a tough problem that an appropriate storage of the loaded RBC has not been founded, which significantly affected the application of erythrocytes in drug delivery and therapeutics. In addition, the equipment and environment of this loaded process *in vitro* must be rigorously controlled to prevent biological contamination which could bring many troubles to the application.

## Conclusion

Protein adsorption resistance is an essential property of drug-loaded carriers in systemic administration. Considerable progress has been made in the surface modification of highly protein-resistant materials to prolong the circulation time in the bloodstream and enhance accumulation of therapeutic agents. Although PEG has been recognized as a desirable protein resistance polymer, the PEG modification significantly interferes with the cellular uptake and endosomal escape of drug-loaded carriers. Therefore, many researches have been done to investigate the usefulness of the other materials such as zwitterionic polymers, which also have been demonstrated to show excellent protein resistance ability and biocompatibility, and are very promising in the development of drug delivery system. However, the zwitterionic materials and RBC are still in the early stage in both conception and practical application. It is expected that protein resistance materials for long-circulating drug delivery system will be widely studied and used in the future development.
